# Chitosan and Its Potential Use as a Scaffold for Tissue Engineering in Regenerative Medicine

**DOI:** 10.1155/2015/821279

**Published:** 2015-10-04

**Authors:** Martin Rodríguez-Vázquez, Brenda Vega-Ruiz, Rodrigo Ramos-Zúñiga, Daniel Alexander Saldaña-Koppel, Luis Fernando Quiñones-Olvera

**Affiliations:** Translational Neurosciences Institute, Department of Neurosciences, University Center of Health Sciences CUCS, Universidad de Guadalajara, 44340 Guadalajara, JAL, Mexico

## Abstract

Tissue engineering is an important therapeutic strategy to be used in regenerative medicine in the present and in the future. Functional biomaterials research is focused on the development and improvement of scaffolding, which can be used to repair or regenerate an organ or tissue. Scaffolds are one of the crucial factors for tissue engineering. Scaffolds consisting of natural polymers have recently been developed more quickly and have gained more popularity. These include chitosan, a copolymer derived from the alkaline deacetylation of chitin. Expectations for use of these scaffolds are increasing as the knowledge regarding their chemical and biological properties expands, and new biomedical applications are investigated. Due to their different biological properties such as being biocompatible, biodegradable, and bioactive, they have given the pattern for use in tissue engineering for repair and/or regeneration of different tissues including skin, bone, cartilage, nerves, liver, and muscle. In this review, we focus on the intrinsic properties offered by chitosan and its use in tissue engineering, considering it as a promising alternative for regenerative medicine as a bioactive polymer.

## 1. Introduction

Currently, regenerative medicine is one of the most popular scientific fields and the future of life sciences, where new technology and today's public health challenges converge.

Regenerative medicine is defined as the association of tissue engineering, stem cells research, gene therapy, and therapeutic cloning, as important strategies for regenerative medicine in the present and future.

Recently, research on biomaterials has pointed to the design, development, and improvement of scaffolds as new drug release systems and bioactive molecules for regenerative medicine [1].

All of this has been possible because of dramatic advances in the field of tissue engineering during the last 10 years, offering potential regeneration of almost all tissues and organs of the human body.

Tissue engineering is an important therapeutic strategy for present and future medicine. Therefore, the goal in tissue engineering is to restore, regenerate, maintain, or improve function in defective tissue or lost tissue due to different disease conditions. This may be possible by using the development of biologic substitutes or by rebuilding structural scaffolds that induce tissue regeneration. Tissue engineering is defined as the use of isolated cells or cell substitutes, tissue inducers, and cells placed on or in a matrix to repair and regenerate tissue [2]. Strategies can be classified into three groups: (1) isolated cell implants or cell substitutes in the body, (2) tissue inducer substances (such as growth factors), and (3) cells placed on or in different matrices or substrates that work as a vehicle or scaffold that induce tissue regeneration [3].

Thus, the focus on tissue engineering points to the use of structures used to repair injured tissue or tissue with structural malformations and also reinforce and, in certain cases, organize regenerating tissue that would work as a scaffolding to restore or regenerate the damaged tissue [4].

## 2. The Scaffolding

Scaffolding is defined as 3D porous solid biomaterials, designed for the following functions: (1) promoting cell-biomaterial interactions, cell adhesion, and extracellular matrix deposits (ECMD), (2) allowing for sufficient transport of gases, nutrients, and regulatory factors to allow for cell survival, proliferation, and differentiation, (3) breaking down at a controllable rate that is close to the regeneration rate of the tissue of interest, and (4) creating minimal inflammation [5].

Biomaterials used as scaffolds in tissue engineering must meet certain requirements or characteristics in order that they can perform the above functions (Table 1) [23]. This scaffolding composed of natural or synthetic materials is commonly used as scaffolds to interact with biological systems to accomplish desirable medical outcomes in modern healthcare, providing alternatives to overcome the limitations and restrictions imposed by the use of autograft and allograft tissues [23].

Polymers are biocompatible biomaterials for application in regenerative medicine and tissue engineering. They can be natural, synthetic biodegradable, and synthetic nonbiodegradable. Among them, the polymers that are mostly used as biomaterials are the natural and synthetic biodegradable ones, which have attracted significant interest because of their flexibility in terms of chemical manipulation and the ability to break down into low molecular weight fragments that can be eliminated or resorbed by the human body [24, 25].

Natural polymers can be considered as the first biodegradable biomaterials used in human clinical conditions [24]. These natural materials, due to their bioactive properties, tend to have greater biological interaction with the cells, which allow them to have better performance in the biological system. Natural polymers can be classified as proteins (silk, collagen, fibrinogen, elastin, cheratin, actin, and myosin) and polysaccharides (cellulose, amylose, dextran, chitin, and glycosaminoglycans) or polynucleotides (DNA, RNA) [26].

Currently, polymers have been widely used as biomaterials for manufacturing medical devices and scaffolds in tissue engineering [27, 28]. In biomedical applications, the selection criteria of polymer materials used as biomaterials are based on certain features such as chemical composition, molecular weight, solubility, shape and structure, hydrophilicity/hydrophobicity, surface energy, water absorption capacity, breakdown, and erosion mechanism. Polymer scaffolds are attracting a great deal of attention due to their unique features, such as the high surface-volume ratio, great porosity on the surface with a very small pore size, ability to control biodegradation, and mechanical properties. They have several biocompatibility benefits, versatility in surface chemistry, and biological properties that are important in tissue engineering application and organ replacement in regenerative medicine [29]. In this context, chitosan has drawn a lot of attention.

## 3. Chitosan Characteristics

**Figure 1:**
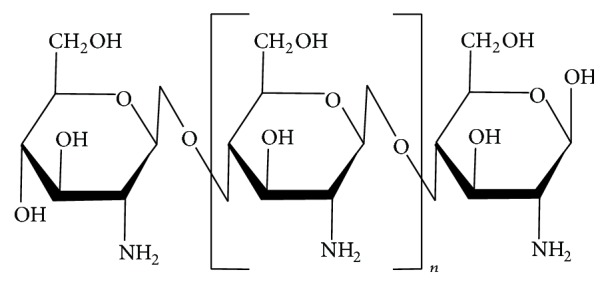
Chemical structure of chitosan [poly-(*β*-1/4)-2-amino-2-deoxy-D-glucopyranose].

Chitosan [poly-(*β*-1/4)-2-amino-2-deoxy-D-glucopyranose] [30] is a copolymer made of D-glucosamine and N-acetyl-D-glucosamine bonds and *β* bonds (1–4) (Figure 1), in which glucosamine is the predominant repeating unit in its structure; it is a derivative of the alkaline deacetylation of chitin, and the glucosamine content is named according to the degree of deacetylation (DD). Depending on the procedure of origin and preparation, molecular weight may vary from 300 kD to over 1,000 kD, with a deacetylation between 30% and 95% in the available commercial preparations. Chitosan has been the best version of the chitin polymer because it is readily soluble in diluted organic acids, thereby having greater availability to be used in chemical reactions [27, 28, 31–33].

Chitosan properties are very much affected by the conditions in which the material is processed because the manufacturing process conditions are the ones that control the resulting amount of deacetylation. The DD in chitosan is a key feature that determines its physical, chemical, and biological characteristics. The DD is determined by the amount of free amino groups in the polymer chain, and this free amino group confers a positive charge to chitosan. The amino group and the hydroxyl group provide functionality, so chitosan turns out to be a highly reactive polysaccharide. The positive charge in chitosan allows for many electrostatic interactions with negatively charged molecules. The processing conditions, as well as the amount of functional groups created by deacetylation, allow for coupling of the groups, having an impact on the crystallinity of chitosan which in turn is directly associated with the ability of chitosan to be soluble in aqueous acid solutions, resulting in one of its main features for processing [34].

Chitosan has many physical and chemical properties conferred by its functional groups (amino NH2 and hydroxyl OH), as well as biological properties coming from its chemical composition. Solubility, biodegradability, reactivity, and absorption of many of its substrates depend on the amount of protonated amino groups in the polymer chain and thus in the rate of acetylated or nonacetylated glucosamine [35, 36]. All of these features make it an attractive option for several applications in science such as food/nutrition, medicine, microbiology, immunology, agriculture, and veterinary medicine [37].

## 4. Physicochemical Properties

### 4.1. pH Dependence and Solubility

Chitosan solubility depends on the distribution of free amino and N-acetyl groups. In diluted acid solutions (pH ≤ 6) the free amino groups are protonated and confer a polycationic behavior and the molecule becomes soluble [4]. From the pka standpoint, similarly, the amino groups (pka 6.2–7.0) are completely protonated in acids with a pka less than 6.2, making chitosan soluble, remaining so until reaching a pH near 6.2, when, at a higher pH (>de 6.5) the amines in chitosan deprotonate and chitosan become insoluble, after which precipitates, such as hydrated gels, are formed. Chitosan is insoluble in water, aqueous solutions, concentrated acids, and common organic solvents, but it is totally soluble when stirred into aqueous solutions such as acetic acid, nitric acid, hydrochloric acid, perchloric acid, lactic acid, and phosphoric acid [36, 38, 39].

### 4.2. Degree of Deacetylation (DD)

The degree of deacetylation (DD) represents the rate of D-glucosamine units with respect to the total amount of N-acetyl-D-glucosamine that makes the chitosan molecule, since this unit is found in the amino group created from the elimination of the acetyl group. A deacetylated chitin over 60 or 70% is already considered to be chitosan. The DD is a structural parameter that determines some physical and chemical properties such as solubility limit in acid solutions (pH 2–6), molecular weight, and mechanical properties (elasticity and traction resistance). The deacetylation process turning chitin into chitosan will transform the acetyl group into a primary amino group, which is more hydrophilic than the preceding molecule; thereby, the DD in chitosan increases the water content in samples taken from chitosan samples, tending to have an impact on the ability to absorb water and limiting the ability to have maximum swelling [40].

The DD also has an impact on biological properties, such as the in vitro and in vivo biodegradation. It has been proven that, at a greater DD (between 84 and 90%), the degradation process is delayed. Highly deacetylated chitosan (over 85%) shows a low degradation index in the aqueous environment and will degrade after a few months, and a lower DD (between 82 and 65%) would lead to a faster degradation. The commercially available preparations have a DD between 60 and 90%. This feature has an impact on some biological properties in chitosan, such as healing capacity, increase in osteogenesis, and a breakdown process by lysozymes in biological systems [41, 42].

The DD plays a key role in cell adhesion and proliferation but does not change the cytocompatibility of chitosan. “In vitro” studies have shown that the lower the DD in chitosan, the lower the cell adhesion in the films. It has been found that keratinocytes attached to the chitosan film may change their adhesion and cell growth depending on their deacetylation degree (DD), but proliferation is not promoted. Thus, DD affects growth of cells in the same way as cell adhesion.

### 4.3. Molecular Weight

Depending on where and how the preparation procedure is done, the molecular weight may change from 300 to over 1000 kD [4]; viscosity and molecular weight are inversely proportional to the degree of acetylation. Therefore, the greater the molecular weight is, the chitosan membranes tend to be more viscous, thus allowing for controlling fluidity in them, an important feature in tissue interaction. Due to its high molecular weight and its lineal nonbranched structure, chitosan is a strong viscosity-building agent in acid mediums and behaves as a pseudoplastic material, where viscosity depends on agitation [43].

It has also been proven that the molecular weight has an indirect effect or is inversely proportional to the swelling capacity and/or hydration of chitosan membranes, and when the molecular weight is greater and the DD is higher, the swelling or permeability is less in chitosan membranes [44].

There is a direct association between molecular weight and DD. These two parameters have a direct effect on the biodegradation process of chitosan, since at a greater molecular weight the degradation process is delayed in “in vitro” as well as “in vivo” systems [45].

After oral administration in mice and rats, the molecular weight decrease in chitosan leads to greater absorption. When low molecular weight chitosan was absorbed, it was found to extend to several inspected organs, that is, liver, kidney, spleen, thymus, heart, and lung and it was also easily metabolized [44, 46].

### 4.4. Porosity

Porosity is a feature in polymers used as scaffolding. Porous scaffolds serve to provide support in tissue engineering because they act as a platform and provide necessary support to physically guide differentiation and proliferation of cells for tissue growth “in vivo” and “in vitro.” The porous scaffolding must be similar to MEC present in tissue, allowing for organized cell growth and neovascularization. For all these reasons, these polymers such as chitosan have been extensively investigated for such applications, specifically for soft tissue replacement because porous scaffolding can retain water in its polymeric structure as well as retaining bioactive proteins [47].

**Figure 2:**
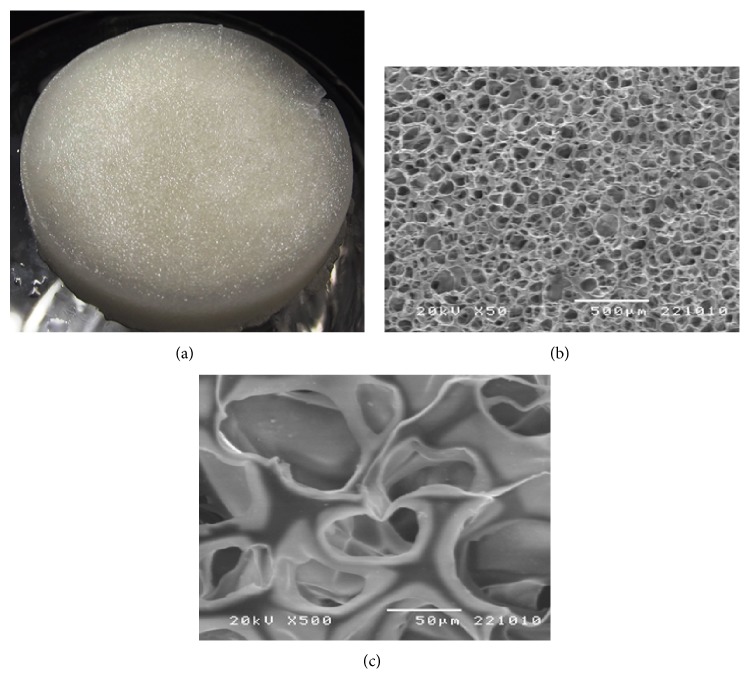
Macroscopic photographs (a) and micrographs (SEM) ((b) and (c)) of porous chitosan scaffold. Micrographs show low and high magnification.

In order for chitosan scaffolding to be used as structural support in tissue regeneration, it should be highly porous so as to have the proper cell proliferation in the action site and also have enough surface area for live cells to accommodate adequately, have the correct pore size so that the growing cells can penetrate and proliferate, and have highly interconnected pore structures that allow cells to grow and to have proper transport of nutrients (Figure 2) [48].

Several methods are used to prepare 3D porous scaffolds, among which we find that of thermally induced phase separation (TIPS) in which temperature is reduced to freezing conditions to induce phase separation in a degradable homogenous polymer. Since chitosan is a polymer, it can produce porous membranes to be used as scaffolds [49].

Porous scaffoldings resulting from the TIPS method can synthetize different structures that contain somewhat different pores with size ranges from 1 to 250 *μ*m and that vary according to temperature and water content. For example, the lower the temperature and the greater the water content, the smaller the pore size; porosity in chitosan membranes has a direct effect on its surface size. Hydrated porous chitosan membranes have been shown to have at least twice more surface size and volume compared to nonporous chitosan membranes, but their elasticity and resistance to traction are ten times smaller than nonporous membranes used as controls [50]. The porous structure can be stabilized adding glutaraldehyde, polyethylenglycol, heparin, or collagen, allowing the structure to become more resistant and to maintain elasticity. These compounds can make chitosan turn insoluble in acid solutions and consequently form closed pore structures [51, 52].

Currently, ideal scaffolding should have 80 to 90% porosity with a pore size of 50 to 250 *μ*m. Its pores should be interconnected so as to provide physical support to cells and guide their proliferation and differentiation, also facilitating neovascularization. The pore sizes recommended for skin scaffolding should be greater than 160 *μ*m, varying between 15–100 *μ*m and 100–200 *μ*m, with a desired 90% porosity to provide the necessary space and enough surface to grow cells and create priority temporary scaffolds for implantation allowing for regeneration or damaged tissue repair [53].

### 4.5. Water Absorption Capacity

When placed in liquid media, chitosan membranes can swell and retain a given water volume absorbed from the medium in their three-dimensional network. In order to be used for biomedical purposes, they should absorb fluid from the body for cell transference, plus they should allow for an adequate distribution of nutrients, metabolites, and growth factors, through extracellular media [47].

### 4.6. Mechanical Properties

Considering the appropriate mechanical properties we can state that chitosan membranes have a disadvantage when used for support in tissue engineering because these membranes are very stiff and brittle; that is, they have low mechanical resistance [23]. So then, in order to optimize resistance and elasticity, crosslinking agents are used with at least two functional reactive groups that allow for making bridges between polymeric chains with formaldehyde, epoxides reacting with polyethyleneglycol, dialdehydes (glutaraldehyde and glyoxal), and starch [54]. In cross-linked hydrogels the polymeric chains are bound by the crosslinking agent, building a 3D network. Their nature will mainly depend on the density or crosslinking degree, named according to the ratio of moles in the agent with the moles in the repeat units of the polymer. Among the reactions to the crosslinks in chitosan with some other materials we can find the aldehyde-amine reaction with polyethylene glycol, which is used because it is hydrophilic, with low toxicity and good biocompatibility. Studies have been conducted showing the effectiveness of the chitosan-DiepoxyPEG (Diepoxy-polyethylene glycol) crosslinking, resulting in an improvement of the mechanical properties of the crosslinked compound [54, 55].

### 4.7. Biological Properties

Chitosan has many beneficial biomedical properties, such as biocompatibility, biodegradability, and no toxicity. Biological activity of chitosan is closely related to its solubility and therefore molecular weight and DD [56].

### 4.8. Biodegradability

The process of biodegradation of chitosan can be through various media both physical (thermal degradation) and chemical (enzymatic degradation); the rate of degradation of the chitosan is inversely proportional to the degree of crystallinity of the polymer and therefore the DD and, thus, can manipulate the degradation rate by controlling the DD which occurs during processing [41, 57].

There is a broad range of hydrolytic enzymes such as lysozyme, which is the primary enzyme responsible for chitosan degradation in “in vivo systems” and that is found in lymphoid human and animal tissue and that can be used to naturally degrade chitosan [58, 59]. Inside the body it leads to the release of amino sugars that can be processed and released by the metabolic system. Chitosan degradation is an important property assuming that the end processes and applications it will ultimately be given can agree with the resulting design [35].

Some of the specific enzymes that degrade chitin and chitosan have a clearly identified structure but their action mechanisms are still unknown. In mammals, these enzymes seem to be completely absent; however, when chitosan is implanted, it will eventually disappear completely after some time and the degradation speed seems to depend on DD [35].

Through enzymatic hydrolysis mechanisms chitosan may be easily depolymerized due to susceptibility *β* bonds (1–4) mediated by different hydrolases including lysozymes, pectinase, cellulases, hemicellulases, lipases, and amylases among others, which means that chitosan has a peculiar vulnerability to other enzymes that are different from chitinases. Its degradation products are oligosaccharides or monosaccharides, natural metabolites of glycosaminoglycans or glycoaminoproteins. Lysozyme is an unspecific proteolytic enzyme common in mammals. It can hydrolyze chitosan, but this action quickly disappears when chitosan has a degree of acetylation (DA) below 30% [41].

When chitosan is fully acetylated, it is totally insensitive to this enzyme. Moreover, it seems that at least three consecutive N-acetylated groups are necessary to be recognized by this enzyme and, in spite of depending on the amine content in chitin and chitosan the unspecific enzymes that degrade these polymers are inactivated, and such degradation can be found in in vivo implants. It has been proven that whatever the circumstances may be, biodegradation of chitosan takes place depending on many and diverse factors, especially the degree of acetylation, molecular weight, degree of crystallinity, water content, and also the shape and condition of the surface on the material, aside from its microstructure [41].

### 4.9. Biocompatibility

Biomaterials should be biocompatible; that is, contact with the body should not result in adverse reactions, so they must be capable of recognizing and cooperate harmoniously with structures and cells of the human body, without producing unspecific reactions [60]. Clinical tests conducted so far have not reported any inflammatory or allergic reactions after implantation, injection, topical application, or ingestion of chitosan in the human body [40]. This is due to the fact that chitosan is made of GlcN and GlcNac that are natural components of mammalian tissues [61].

There are studies that support the biocompatibility of the material and the direct relationship of this property with its DD material used. In the first reports that determined toxicity, none of the materials were chitosan films using a standard “in vivo” toxicity tests to assess their safety [62]. The biocompatibility of chitosan films has also been shown with different DD; studies where a model of subcutaneous implantation in rats is applied showed that the films of chitosan with DD between 69 and 74% induced a relatively acute inflammatory reaction by rapid biodegradation, with almost complete resorption after 4 weeks of implantation; DD films with high (between 74 to 90%) resulted in a mild inflammatory reaction in tissue degradation rate and a slower rate. This is in agreement with the well-known fact that rapidly biodegradable biomaterials elicit an acute inflammation reaction due to a significantly large production of low-molecular-weight compounds within a short time. Therefore it was determined that films with ≥ 84% DD showed reaction to softer tissue because these were degraded more slowly [45].

### 4.10. Non Toxic

Several studies have proven that, so far, clinical tests conducted with chitosan have not reported adverse inflammatory or allergic reactions when used in tissue engineering or as a vehicle in drugs, nor after implantation, injection, or topical application in the human body or for oral application. This property is due to the fact that chitosan is made of GlcN and GlcNAc, natural components of mammalian tissue [41, 42, 63].

## 5. Cytocompatibility

A wide number of cells have been successfully cultured on 2D and 3D chitosan matrices envisaging cell-based regenerative therapies, among them keratinocytes, chondrocytes, osteoblasts, hepatocytes, and Schwann cells [40, 64–68]. Some studies found that the DD was an important parameter affecting cell adhesion, by promoting high adhesion. This effect was reported for a number of anchorage-dependent cells, such as keratinocytes, fibroblasts, dorsal root ganglion neurons, and Schwann cells [69–71].

In other studies the effect of DD concerning behavior of the osteogenic cells and chitosan films was investigated in porous matrices, using DD in the range of 96–51%. These studies revealed a trend that determines that there is an increase of cell adhesion related to an increase in the DD, and the differences showed that a DD greater than 91% can be critical in terms of osteogenic response from chitosan [72, 73].

## 6. Nonimmunogenic

Chitosan and its oligomers stimulate macrophage activity increasing nitric oxide, reactive oxygen species, TNF-*α*, interferon, and IL-1, as well as TGF-*β*1 and PDGF. However, since there are no proteins and lipids in its structure, it is not possible to develop specific antibodies against it, unless it is coupled with other substances such as albumin. The conclusion from all the studies on the subject indicates that chitosan is hypoallergenic and only transiently stimulates the immune system because when impregnated with tissue fluids in the receptor body it ultimately becomes biotolerated and metabolized [41, 63, 74].

## 7. Antimicrobial and Antifungal

Chitosan inhibits growth of many types of fungi, yeasts, and bacteria. In solutions made of acid dilutions the positive loads in them interact with the negatively charged residues of macromolecules on the cell surface of microorganisms, supposedly competing against the Ca+2 for the electronegative sites in the membrane, but without conferring dimensional stability, compromising the membrane integrity and making it weak [75]. Among the antimicrobial effects of chitosan are those related to* Candida albicans*,* Enterobacter cloacae*,* Enterococcus faecalis*,* Escherichia coli*,* Klebsiella pneumoniae*,* Pseudomonas aeruginosa*,* Staphylococcus aureus*, and* Streptococcus pyogenes*.

This property is of special significance because it has been proven that the antimicrobial agents such as bandaging materials and dressings generally lead to cytotoxicity, delaying the healing process, or leading to pathogen resistance. In the case of chitosan, since the antimicrobial effects come directly from the membrane, there is almost no need to use antibacterial substances or change bandages, the implants themselves, or dressings when they are applied [63].

## 8. Tissue Repair and Regenerative Medicine

In regenerative medicine applications of biomaterials for tissue repair and regeneration include their use as orthopedic implants and, as bone fillers, adhesives for tissue repair and the use of scaffolds for tissue engineering; the latter are used for repair and/or regeneration of skin, bone, cartilage and nerve tissues, since these tissues have been the focus of greater research in regenerative medicine. This involves the use of chitosan as a scaffolding material or as an analog or extracellular matrix (ECMD), which works as support for the regeneration of damaged tissue (Table 2) [6–22, 76].

The popularity of chitosan for tissue repair and regeneration is due to the fact that it can be easily processed and manufactured in a variety of forms including fibers, films, sponges, and hydrogels. This provides the ability to mimic the shape of the receiving tissue or biomaterial tissue interface. Moreover, the similarity of its chemical structure to some polysaccharides and ECM constituents offers the possibility of being chemically modified to adapt structurally and functionally to the host tissue, due to its previously described properties that allow for the ability to regenerate primary tissue cells and even stem cells. Thus its potential is to be used in regenerative medicine [4, 33, 77, 78].

## 9. Chitosan and Tissue Engineering

### 9.1. Skin, Nerves, and Soft Tissues

The generation of scaffolds with porous structures is important in the engineering of epithelial and soft tissues. Chitosan can be manufactured in a porous structure to allow for cell seeding. This space created by the porous structure allows for cell proliferation, migration, and the exchange of nutrients. In addition, the controllable porosity of chitosan scaffolds is beneficial to angiogenesis, which is fundamental in supporting the survival and function of the regenerated soft tissues [50, 79]. Chitosan scaffolds have shown both cytocompatibility in vitro and biocompatibility in vivo. Generally, chitosan evokes only a minimal foreign body reaction in vivo, and implanted chitosan scaffolds seldom induce chitosan-specific reactions [32].

Due to the fact that some chitin-based biomaterials do not provide a friendly interface for cell adhesion of some specific tissue types, other biomaterials, such as collagen or fibronectin with tissue-specific binding sequence, should be blended with chitosan to produce scaffolds with higher cell affinity. Also, chitosan is blended with other biomaterials to create scaffolds that are more appropriate for directing the desired cell behaviors and to mechanically strengthen the tissues engineering of tissues such as the skeletal system [80, 81]. The biological activity beneficial to tissue regeneration can be introduced through the entrapment of bioactive agents in the scaffolds through physical adsorption [6]. For example, trimethylated chitosan has been reported to be efficient in gene transfection without increasing cytotoxicity [82].

Specifically speaking about the difference in tissues, there is evidence that chitin-based materials support neuronal growth. In addition, many different substrates and bioactive molecules have been added into chitin-based scaffold to increase their affinity with nerve cells [83]. A chitosan tube immobilized with laminin peptides can facilitate proximal nerve sprouting and regenerate axon bridging [84]. In 2004, a study showed that chitosan fibers supported the adhesion, migration, and proliferation of Schwann cells, which allowed for axonal regeneration in the peripheral nervous system [68]. Whenever there is a peripheral nerve lesion, the current standard treatment is to use an autologous nerve graft to bridge the neural gap and facilitate nerve regeneration and reconnection; however, since there have been frequent and severe complications, several attempts have been made over the last decades to overcome this problem by using different biomaterials but functional recovery is still far from being acceptable [85]. Nevertheless, different scaffolds were fabricated in a study by cross-linking chitosan with acetic acid and chitosan with *γ*-glycidoxypropyltrimethoxysilane, which would later be cultivated with N1E-115 cells, derived from mouse neuroblastoma C-1300. The resulting hybrid membranes presented good cytocompatibility besides the fact that, when cultured in the presence of dimethylsulfoxide (DMSO) or cyclic AMP (cAMP), they show characteristics from neural cells, such as ceased multiplication, extensive neurite outgrowth, and polarization of cellular membranes, being able to locally produce and deliver nerve growth factors, essential in the reconstruction of peripheral nerve lesions. In vivo studies suggest that these chitosan-based membranes show promising results regarding its applications in peripheral nerve engineering due to their porous structure, their chemical modifications, and high affinity to cellular systems [85]. These modifications make chitin-based materials more diverse and functional for soft tissue regeneration. In the same manner, it was found that in the regeneration of ligaments, chitosan-hyaluronic hybrid polymers can provide appropriate environments for cellular adhesion, proliferation, and extracellular membrane (ECM) production, as well as facilitating the biological effects of seeded cells [6].

For vascular tissues, in order to mimic the morphological and mechanical properties of blood vessels and improve long-term patency rates, collagen has been crosslinked with chitosan to generate a tubular scaffold. This biocompatible scaffold proved to have desirable porosity and pliability, enhanced cell adhesion, proliferation, and ECM production [86, 87]. In addition to vascular applications, chitosan/collagen blended scaffolds have also been employed in adipose tissue regeneration. When adipocytes were seeded, the in vitro cytocompatibility and in vivo biocompatibility of scaffolds were confirmed experimentally [88].

Chitosan also has a potential use in skin repair and regeneration subsequent to injuries or burns. A study was performed in which chitosan was cross-linked with silica particles (SiO2), used as a porogen agent and the extractions from the developed membranes demonstrated no cytotoxicity against L-929 cells 24 hours after the culture. In addition, the macroporous membrane exhibited excellent cellular adhesion and proliferation after 24 and 48 hours of culturing, which is why the developed scaffold might be adequate for skin tissue engineering [89]. Chitin-based materials have also demonstrated their potential in maintaining and inducing cell phenotypes used in culturing melanocytes, corneal keratinocytes, and skin keratinocytes [90, 91]. Even in the salivary gland, the morphogenetic efficacy of mesenchyme-derived growth factors is dramatically augmented with the assistance of chitosan. The effects of epithelial morphogenetic factors, such as fibroblast growth factors 7 (FGF7), fibroblast growth factor 10 (FGF10), and hepatocyte growth factor (HGF), have been upregulated in the presence of chitosan [88].

Furthermore, chitosan use in the design of new tissue adhesives was motivated by the fact that it can bind to collagen due to hydrogen bonding and polyanionic–polycationic interactions [88]. There is evidence that hydrogels and meshes of chitosan cross-linked with other biomaterials are useful in the prevention of postoperative abdominal adhesions. A study was developed in which they used thermosensitive hydroxybutyl chitosan (HBC) in a rat side-wall defect-cecum abrasion model for prevention of postoperative abdominal adhesions. HBC is a new derivative of chitosan whose main character is the intelligent response to changing temperature. HBC demonstrated antiadhesive activity as well as being easy to handle during the operation. Therefore, it may be effective in prevention of postoperative adhesions [92]. Another protocol was performed where they compared 3 different types of meshes: Dynamesh-IPoM mesh, a simple polypropylene mesh, and a polypropylene/chitosan mesh. The results were that the polypropylene/chitosan mesh proved to be the least irritating for the recipient's tissue as well as surrounding tissues, as evidenced by the lowest rate of inflammatory reaction within the connective tissue, which guarantees the implant acceptance and the least extensive adhesion to internal organs, and thus the lowest rate of complications [93]. Finally, there was another protocol in which they determined that a chitosan-gelatin modified film modified chitosan film is effective on preventing peritoneal adhesions induced by wound, ischemia, and infection, but the effect is not apparent in foreign body-induced adhesion [94].

Due to the properties that chitosan has shown in reduction and prevention of postoperative intraperitoneal adhesions, it is also widely being studied for its use in repair and regeneration of the abdominal wall in ventral hernias. A study was conducted to investigate the feasibility of using silk fibroin and chitosan blend scaffolds for ventral hernia repair in guinea pigs [20]. This blended scaffold was compared to a biodegradable human acellular dermal matrix and a nonbiodegradable polypropylene mesh. The investigators concluded that the silk fibroin and chitosan blend scaffold, unlike the mesh and the matrix, showed tissue remodeling in all 3 dimensions, with seamless integration at the interface with adjacent native tissue, the repair sites remained intact, and their mechanical strength was similar to that of the native abdominal wall. Additionally, the scaffold promoted the deposition of new extracellular matrix, uniform vascularization, and cellular infiltration in the repair sites, which contributed to the increase in mechanical strength of the regenerated tissue. Thus, this scaffold is potentially useful in reconstruction and regeneration of the abdominal wall [20]. Furthermore, due to its utility in ventral hernia repair, it might also have a potential use in inguinal hernias and other types of herniation. It might even be useful in the repair of certain congenital defects such as omphalocele or gastroschisis, although to date there are still no apparent models that prove its effectiveness for application in humans for these types of defects.

Specifically speaking about intestinal tissue, its engineering is an emerging field due to a growing demand for intestinal lengthening and replacement procedures secondary to massive bowel resections [95, 96]. Intestinal transplantation is a common treatment but its limitation resides in the high incidence of rejection, availability of donor organs, and the size of the donor graft. The biocompatibility of chitosan was investigated by growing rabbit colonic circular smooth muscle cells on chitosan-coated plates [95]. The cells maintained their spindle-like morphology and preserved their smooth muscle phenotypic markers. Tubular scaffolds were manufactured with central openings composed of chitosan and collagen in a 1 : 1 ratio. Concentrically aligned 3D circular muscle constructs were bioengineered using fibrin-based hydrogel seeded with the colonic circular smooth muscle cells from the rabbit. The muscle constructs contracted in response to acetylcholine (Ach) and potassium chloride (KCl) and they relaxed in response to vasoactive intestinal peptide (VIP). These results demonstrate that chitosan is a biomaterial possibly suitable for intestinal tissue engineering applications [95].

In conclusion, it is safe to say that chitosan has great potential in applications for soft tissue engineering, whether it is used for wound closure or for its potential use in generating specific tissue grafts. Nonetheless, there is still much research to be done in terms of their properties and formation of scaffolds. Next generation scaffolds should be able to carry many different bioactive factors and release them in specific order. To this end, decisions on how to control the separate loading capacity, kinetics of drug release, and rate of substrate degradation are the major challenges to be faced [88].

## 10. Potential Applications Supported by Its Biological Activity

### 10.1. Hemostatic Properties

Chitosan is capable of promoting platelet adhesion by initializing a cascade of intracellular signaling which activates glycoproteins IIb/IIa as well as thromboxane A2/ADP, increasing platelet spreading and strengthening the stability of adhesion [97]. Chitosan is available in different presentations, including films, fibers, and hydrogels, and each one of them offers specific advantages in terms of absorption depending in its therapeutic use.

Several studies have proven efficacy of chitosan as a hemostatic agent. However, it has been reported that the hemostatic mechanisms of chitosan are separate from the classic coagulation cascade [62]. The hemostatic effect in chitosan is achieved from the direct interaction with platelets, mainly in alpha granules. The intracellular signaling induces PDGF-AB and TGF-*β*1 release. An increased rate in the PDGF-AB has been found, as much as up to 130%, with the use of chitosan compared to a control group [98]. Chitosan can be used in medical and surgical procedures by direct application on bleeding surfaces, using several presentations such as powder, solutions, coatings, films, hydrogels, compounded filaments, and more. Nevertheless, its clinical use will depend on the application technique used as well as the type of wound that it is applied on. Many investigators have described the hemostatic applications for chitosan; however, different presentations have been used in each study; that is, why results should be analyzed according to the different groups, depending on the physical form of the material [99].

### 10.2. Liver Repair Application

There are three crucial factors for successful use of chitosan in surgery: it must be placed in the intra-abdominal cavity, it must have good bonding to the surface of the lesion, and it must be able to maintain proper hemostasis.

A study compared the effectiveness of a freeze-dry chitosan graft versus the use of sponge gauze in a venous bleeding due to a severe liver lesion in a pig model. The animal model involves extensive vascular damage, as well as damage to the liver parenchyma. Several vascular lesions of approximately 1 cm in diameter were made. The outcome reported with the chitosan graft had less blood loss (*p* < 0.01) compared to the group of sponges (264 mL and 2,879 mL, resp.) [100].

In further investigations the hemostatic effects of chitosan as a solution have been previously analyzed. The results of a lingual incision in a heparinized animal model according to the evaluations with electron microscopy concluded that the incisions treated with chitosan showed a disturbance in the morphology of red cells, as well as an unusual affinity among the red cells. It was reported that the red cell fractions that interacted with chitosan reduced bleeding in 60%, reaching hemostasis at 800 *μ*g/mL [99].

### 10.3. Healing Properties

In another study it was found that, by means of chitosan hydrogel application on skin wounds in diabetic mice, the wound shrinking speed improved and wound closure was significantly faster. The chitosan hydrogel combined with fibroblast growth factor type 2 was seen to accelerate the closing process even further. Histology examination showed that the combination of chitosan and fibroblast growth factor type 2 fostered the formation of granulation tissue, capillary network, and epithelialization [101].

The regenerative properties of chitosan are based on a matrix building capacity that is adequate for growth and activation of macrophages and proliferative cells in three-dimensional tissue. A comparative study between wounds treated with chitosan and a control group treated only with saline solution was conducted in a dog animal model. The wounds were clinically assessed throughout the study and inspected histologically once the animal was euthanized. Clinically, complete healing was achieved in the chitosan-treated group after three weeks, while in the control group it took four weeks. A complete repair of epidermal cells with a keratin layer associated with connective tissue proliferation was seen. In the chitosan group a collagen network of fibers produced by fibroblasts was found, which surrounded the neovasculature of the wound, while in the control group hyalinosis of subcutaneous tissue occurred [102].

### 10.4. Chitosan Composites for Bone and Cartilage Regeneration

Chitosan composites have been synthesized for hard tissue regeneration, as in the case of bone and cartilage.

Evaluation of chitosan composites for bone tissue regeneration is based on physicochemical and biological characterizations. Physicochemical characterization comprises the study of homogeneity, purity, percentage composition, chemical bonding, thermic stability, mechanical tests, and incubation on simulated body fluid [103, 104]. On the other hand biological evaluation includes the test in in vitro culture cells (MC-3T3, hFOB, MG63, and bone marrow stem cells) [105–108]. These assays evaluate composite cytocompatibility and cytotoxicity, in addition to its intinsic capacity of induction of cellular differentiation [109]. Finally the efficacy in the treatment of surgical defects on animal models is evaluated [21, 107, 110].

In the first place, chitosan as a matrix allows for biocompatibility of an implantable material, and in the second, it allows for the interaction or combination with inducer materials for tissue regeneration. In this sense, the use of chitosan composites for tissue regeneration in experimental models is a promising strategy for treatment of skeletal and joint diseases [76, 111, 112].

In the case of bone tissue engineering, chitosan matrices have been combined with osteogenic materials, like hydroxyapatite [103, 108, 109, 111], calcium phosphate and sulfate [113, 114], and others [115–117]. The purpose of the combination of those biomaterials is to obtain organic and inorganic composites that simulate the bone structure [118].

The evaluation of chitosan composites in osteoblast culture is an important factor to identify its biocompatibility. In in vitro assays with the MC3T3 cell line, which comes from calvarial murine osteoblasts, cross-linked membrane of chitosan with tripolyphosphate showed the same values in MTT assay of cell viability compared to controls; results were observed in composites compound of chitosan with calcium phosphate, and chitosan with the release of bone morphogenic protein type-2, concluding that those membranes are biocompatible with osteoblasts. Additionally it has mechanical properties that make it a good implantable composite in bone defects [113, 119].

When bone tissue is damaged by trauma, cancer, or infection, a source of autogenous bone tissue or replacement materials is needed to regenerate the compromised tissue. Experimental animal models are a good resource to understand how biological and pathological conditions participate in the healing of bone tissue. In this sense, chitosan composites can be used to induce bone healing and regeneration.

Chitosan composites have been tested in bone defects in experimental models successfully for bone regeneration. Chitosan hydrogel, gelifiable by blue light, was used for BMP-2 release and showed good bone regeneration in a femoral defect in rat [120]. Similar results were observed with the use of a lyophilized porous membrane, a compound of chitosan and hydroxyapatite, in a calvarial defect in rat, the composite membrane filled up the defect as compared to a control, in addition, the presence of osteogenic markers was more abundant in the experimental group [121].

Osteogenesis is the process where osteoblast cells proliferate from mesenchymal cells and deposit extracellular matrix; at the end of the bone defect these cells differentiate into mature osteocytes. All of this tissue process includes the expression of multiple bone markers and enzymes involved in cell maturation and bone calcification.

Chitosan/nanohydroxyapatite composites have been more relevant for tissue engineering, because of its ability to induce a good proliferative response in osteoblasts, and in a tibial defect in a rabbit it showed good bone regeneration at 8 weeks seen by microcomputarized tomography [122].

Joint defects are common in elderly people, caused by rheumatoid arthritis and dehydration of cartilage tissue in the entire body or by the lifting of heavy loads with the corresponding joint wear, leading to the total dependence of joint replacement therapy.

In the case of cartilage tissue regeneration, chitosan composites have been designed to form hydrogels. These composites allow for the inclusion of cells and molecules for cartilage regeneration. The most combined inducer agent of cartilage regeneration is collagen type II, this is the main protein in cartilage tissue, and it enhances the adhesion and formation of clusters of chondrocytes in vitro [15, 123, 124], a requirement for cartilage regeneration. Collagen II and chondroitin sulfate in chitosan hydrogels stimulate chondrocytes attachment in a blue light gelification composite, it also induces the mesenchymal stem cells differentiation to chondrocytes in vitro [125], and similar results have been observed with chitosan hydrogel with alginate and fibroin. In order to follow the seeding cells on chitosan composites, the next step in regeneration is the formation of functional tissue; collagen II expression by chondroblasts and chondrocytes is a determinant factor in cartilage formation, and it has been observed in the glycerophosphate-chitosan hydrogel with silk fibrils, where chondrocyte phenotype is maintained for the expression of glycosaminoglycans and type II collagen in vitro [126], as obtained with alginate and fibroin in chitosan hydrogels [127]. These findings suggest that chitosan can support the addition of an inducer material and also is a biocompatible material for cartilage tissue engineering. Extracellular matrix deposition is a key factor to recognize biocompatibility and normal cell function, in addition a 3D matrix for growth tissue by proliferation and differentiation of precursor cells. In the case of chondroblast and chondrocytes, production of collagen II is a functional tissue determinant, and this has been found in glycerophosphate-chitosan hydrogel and silk fibrils, which stimulates the production of collagen II and glycosaminoglycans by chondrocytes in vitro [126].

The polylactide acid-chitosan membranes with collagen provide a laminate matrix with mechanical properties similar to cartilage, but in addition they have worked as a support for chondrocytes from rabbit cartilage [128], and this opens the possibility to use chitosan composites in the regeneration of cartilage defects [21], as in the case of arthritis or joint cartilage damage from aging.

A biomaterial combination of chitosan with polycaprolactone [112, 124, 129], silk fibrils [126], genipin [15], chondroitin sulfate [130], and polyester [14] has been designed with the purpose of obtaining composites with properties that resemble those of cartilage, and it also provides a proper environment for extracellular matrix deposition, cell viability, and differentiation.

This perspective allows for understanding the potential properties of chitosan composites in hard tissue regeneration. In summary, chitosan composites provide physical and chemical and mechanical support, cell attachment, proliferation, and differentiation, with the corresponding biocompatibility to induce the bone and cartilage tissue regeneration.

## 11. Conclusions

Regenerative medicine is facing new challenges in the way to induce tissue repair in live tissue. Advances have led to the availability of bioactive compounds for damaged tissue. Such compounds must have a regenerative effect and foster wound repair, with the least possible morbidity and with high biocompatibility conditions. Chitosan polymers have been proven to serve as scaffolds that induce tissue regeneration, but beyond that, they are considered to be an ideal polymer for making bioactive compounds [131]. This is possible because there is a potential synergy in their byproducts, when combined with growth factors and stem cells, either of mesenchymal origin or neural origin. Additional studies should try to confirm the translational issues on the role of bioactive polymers and their real impact on regenerative medicine.

## Figures and Tables

**Table 1:** Characteristics that must contain a biomaterial for use in tissue engineering and regenerative medicine.

Characteristics	Description of the characteristic
Biocompatibility	They must be accepted by the receptor and must not lead to rejection mechanisms because of its presence

Absorbability and degradability	Absorbable, with controllable degradation and resorption rate to be the same as the in vitro and in vivo cell/tissue growth

Not to be toxic or carcinogenic	Its degradation products cannot cause local or systemic adverse effect on a biological system

Chemically stable	Chemical modifications not being present in a biological system implant or biodegradable in nontoxic products, at least during the scheduled time to regenerate tissue

Chemically adequate surface	To have a chemically adequate surface for cell access, proliferation and cell differentiation

Adequate resistance and mechanical properties	Resistance and mechanical properties, superficial characteristics, fatigue time, and weight, according to the receptor tissue needs, as well

The proper design, size, and shape of the scaffolding	Which allows having a structure with properties according to the needs of the receiving tissue to regenerate or repair.

**Table 2:** Applications of chitosan-based scaffolds for tissue engineering.

Chitosan combination	Scaffold obtained	Experimental model	Tissue application	Reference
Chitosan + hyaluronan	Hybrid polymer fiber	Fibroblasts from patellar tendon of Japanese white rabbit	Ligament	[6]

Collagen-chitosan + fibrin glue	Asymmetric porous scaffold	Human dermal fibroblasts and keratinocytes	Skin	[7]

Chitosan + alginate	Polyelectrolyte multilayer film	C2C12 myoblasts	Muscle	[8]

Chitosan + aloe vera	Blended membrane	Bovine articular chondrocytes and mesenchymal stem cells	Skin	[9]

Chitosan alone	Membrane	Embryonal submandibular gland cells	Salivary gland	[10]

Chitosan + layer of chitosan/gelatin	Sandwich tubular scaffold	Vascular smooth muscle cells from rabbit aorta	Blood vessel	[11]

Genipin-crosslinked chitosan, chitosan-nanohydroxyapatite	Framework	Human periodontal ligament tissue, periodontal ligament stem cells	Bone	[12]

Chitosan + collagen	Hydrogel	Epididymal fat pads cells, and subcutaneous pocket of male Lewis rat	Adipose tissue	[13]

Chitosan + polyester	Compressed porous disc	Bovine articular chondrocytes	Cartilage	[14]

Chitosan + collagen + genipin	Crosslinked porous membrane	Rabbit articular chondrocytes	Cartilage	[15]

Chitosan + chondroitin sulphate	Bidimensional glass surfaces or 3D packet of paraffin	Bovine articular chondrocytes and human mesenchymal stem cells culture	Cartilage	[16]

Chitosan + adipose-derived stem cells	Tube nerve conduit	Male, Sprague-Dawley rats sciatic nerve transection	Nerve	[17]

Chitosan alone	Tube	Male, beagle dogs phrenic nerve resection	Nerve	[18]

Chitosan alone	Viscous solution and a monolayer rigid physical hydrogel	Female minipigs third-degree burns	Skin	[19]

Chitosan + silk fibroin	Thin blended film	Female guinea pigs ventral hernia	Muscle	[20]

Chitosan + *β*-sodium glycerophosphate + hydroxyethyl cellulose	Hydrogel	Male and female sheep articular defect	Cartilage	[21]

Chitosan + calcium phosphate cement	Chitosan microspheres inside cement paste	Male rabbit femoral defect	Bone	[22]

## Abbreviations

DD: Degree of deacetylation
TIPS: Thermally induced phase separation
ECMD: Extracellular matrix deposits
ECM: Extracellular membrane.
